# Effect of a single immersion in cold water below 4 °C on haemorheological properties of blood in healthy men

**DOI:** 10.1038/s41598-024-58731-2

**Published:** 2024-04-12

**Authors:** Aneta Teległów, Hatice Genç, Iwona Cicha

**Affiliations:** 1 Department of Health Promotion, Institute of Basic Sciences, University of Physical Education in Krakow, 31-571 Kraków, Poland; 2 Cardiovascular Nanomedicine Unit, Section of Experimental Oncology and Nanomedicine (SEON), Department of Otorhinolaryngology, Head and Neck Surgery, Universitätsklinikum Erlangen, Friedrich-Alexander Universität Erlangen-Nürnberg, Erlangen, Germany

**Keywords:** Blood rheology, Cold water immersion (CWI), Erythrocytes, Endothelial cells, Low temperature, Cell biology, Health care

## Abstract

Cold water immersion (CWI) involves rapid cooling of the body, which, in healthy individuals, triggers a defence response to an extreme stimulus, to which the body reacts with stress. The aim of the study was to determine the effect of CWI on hemorheological blood indicators. The study group consisted of 13 young males. Blood samples were collected before and after CWI. The assessed parameters included the complete blood count, fibrinogen, hs-C-reactive protein (CRP), proteinogram, and blood rheology factors, such as erythrocyte elongation index (EI), half-time of total aggregation, and aggregation index. Additionally, the effect of reduced temperature on primary human vascular endothelium was investigated in vitro. CWI resulted in the decrease of body temperature to 31.55 ± 2.87 °C. After CWI, neutrophil count and mean corpuscular volume (MCV) were significantly increased in the study group, while lymphocyte count was significantly decreased. Significantly higher levels of total blood protein and albumin concentration were detected after the immersion. Among hemorheological characteristics, erythrocyte EIs at shear stress values ranging from 2.19 to 60.30 Pa were significantly lower after CWI. No significant changes in other rheological, morphological or biochemical parameters were observed. In vitro, human umbilical vein endothelial cells responded to 3 h of temperature decrease to 25 °C with unchanged viability, but increased recruitment of THP-1 monocytic cells and changes in cell morphology were observed. This was the first study to evaluate the effect of single CWI on rheological properties of blood in healthy young men. The results indicate that a single CWI may increase blood protein concentrations and worsen erythrocyte deformability parameters.

## Introduction

The beneficial impact of local or systemic cooling and cooling therapies on the human body has been known for a long time. Regular cold water swimming was shown to induce physiological, organ-specific and systemic defence responses that are advantageous and effective in maintaining, or restoring, homeostasis in the human body. Winter swimming is a short-term bath in the cold water of a lake, sea, or other reservoir, usually practised in a group during the autumn and winter season. It enhances immunity, improves the thermoregulatory system, supports the cardiovascular function and blood supply to the skin^1–8^. Notably, winter swimming has also been demonstrated to have a positive impact on human wellbeing ^9^, including the course of depressive disorders^10^. Regular winter swimming can also add to the subjective reduction of body fatigue and soreness. Moreover, cold baths have been shown to improve post-workout recovery^11^. Regular winter swimmers exhibit greater tolerance to low temperatures^12^, owing to a number of adaptive mechanisms^2^. Regular exposure to low temperature furthermore exerts analgesic and anti-oedema effects, improves antioxidant status, reduces inflammation, and positively influences the function of the neuromuscular system^2,5,13^. As reported by Wesołowski et al.^14^, repeated winter swimming helps to efficiently eliminate lipid peroxidation products from the bloodstream. Concerning the inflammatory factors, no significant changes in IL-6, immunoglobulins (IgG, IgM, IgA), or C-reactive protein (CRP) were observed in the studies by Siems et al.^15^ and Jansky et al.^16^ after repeated CWIs. This implies that non-infectious stressful stimuli, such as repeated CWI, which increases the body metabolic rate due to shivering, activate the immune system to a minor extent. According to Knechtle et al.^5^, various hormones (such as catecholamines, insulin, thyroid-stimulating hormone (TSH), adrenocorticotropic hormone (ACTH), as well as cortisol) respond to cold-related stress. However, no statistically significant changes in cortisol or creatine kinase in regular winter swimmers were observed in a study by Teległów et al.^17^. Further, the study confirmed that winter swimming did not alter the biochemical indicators related to the renal and hepatic profiles, which is most likely due to a long-term adaptation to the respective conditions.

In contrast to regular winter swimming with its cold-conditioning effects, a single CWI involving very rapid cooling of the body represents an extreme stress stimulus, that may trigger a defence response. There are very little data, however, on the effects of a single immersion in cold water below 4 °C on the rheological factors. The main objective of the present study was to determine the effect of a single CWI performed by young males on hemorheological, morphological and biochemical parameters, which include blood count, fibrinogen, proteinogram, as well as erythrocyte deformability and aggregation indices. Furthermore, we investigated the in vitro effects of cold temperature on the primary cells of human vascular endothelium.

## Materials and methods

### Participants

The study involved 13 male subjects, physiotherapy students at the Faculty of Physical Rehabilitation, University of Physical Education in Krakow. Healthy individuals aged 21–25 years who did not practise winter swimming were included in the research programme after obtaining doctor’s consent and after physiotherapist consultation. The participants were free of any chronic diseases including diabetes, heart disease, metabolic disorder, endocrine disorder. Smoking, active infections, and neoplasms constituted further exclusion criteria. The participants, fully acquainted with the study details, provided their written consent to take part. All the applied procedures followed the Declaration of Helsinki with its amendments^18^.

### Experimental set-up

The subjects underwent an immersion in a tub of cold water at a temperature of less than 4 °C. The tub was placed in a refrigerator truck (length: 5 m, mass: up to 3.5 t), with the air temperature of – 15 °C. The immersion time equalled 2–3 min. The tub was made of weather- and water-resistant thermally modified wood. Prior to the immersion, the volunteers were trained by multiple Guinness World Record holder Valerjan Romanovski, who supervised the immersion of the study group. Blood pressure, heart rate, and body temperature were measured before entering and immediately after exiting the tub.

Fasting blood in a volume of 10 ml was collected from an ulnar vein in the Blood Physiology Laboratory of the Central Research and Development Laboratory, University of Physical Education in Krakow, by a qualified nurse, before entering and within 7 min after exiting the tub, into Vacuette EDTA K2 tubes and into clot activator tubes. The morphological, hemorheological and biochemical blood indicators listed below were assessed on the day of sampling in the above-mentioned laboratory and in the Diagnostyka S.A. laboratory in Krakow, Poland.

#### Morphological analyses

The morphological evaluation of blood before and after CWI was carried out using an ADVIA 2120i analyser (Siemens Healthineers, Erlangen, Germany). The tested parameters included white blood cell count (WBC) [× 10^9^/l], neutrophil count (NEU) [× 10^9^/l], lymphocyte count (LYM) [× 10^9^/l], monocyte count (MON) [× 10^9^/l], eosinophil count (EOS) [× 10^9^/l], basophil count (BASO) [× 10^9^/l], red blood cell count (RBC) [× 10^12^/l], haemoglobin concentration (HGB) [g/dl], haematocrit (HCT) [%], mean corpuscular volume (MCV) [fl], mean corpuscular haemoglobin (MCH) [pg], mean corpuscular haemoglobin concentration (MCHC) [g/dl], red blood cell distribution width with standard deviation (RDW-SD) [fl], reticulocyte count (RET) [× 10^9^/l], platelet count (PLT) [× 10^9^/l], mean platelet volume (MPV) [fl], procalcitonin concentration [%], and platelet distribution width (PDW) [fl].

#### Rheological assessments

The blood rheology parameters, such as erythrocyte aggregation (aggregation index (AI) [%], amplitude and total extent of aggregation (AMP) [arbitrary units], half-time of total aggregation (T_1/2_)[s]) and deformability (EI, elongation index) were measured with the Laser-Assisted Optical Rotational Red Cell Analyzer (Lorrca) MaxSis (Lorrca^®^, RR Mechatronics, the Netherlands) using the method described by Hardeman and Baskurt^13,19^. Mean EI values were determined at the shear stress values of 0.30–60.00 Pa, using the automatic analysis function of Lorrca system, which allows assessing erythrocyte deformability as a function of shear stress and erythrocyte aggregation.

#### Blood protein analysis

Fibrinogen concentration (FIBR) [g/l] was determined with a BCS Siemens coagulation analyser. Protein electrophoresis was carried out using a Cobas c 311/511 analyser in the Roche/Hitachi systems. Concentrations of albumin (A) and other fractions (alpha-1 globulin, alpha-2 globulin, beta-1 globulin, beta-2 globulin, gamma globulins) were determined on the basis of total protein concentration and electrophoretic fraction percentages resulting from the analysis of electropherograms, obtained upon separating serum proteins by capillary electrophoresis (Minicap, Sebia). Albumin-globulin ratio (A/G) was calculated. High sensitivity CRP (acute phase protein) concentration [mg/l] was assessed using the immunonephelometric method, reagent kits, and the BN ProSpec nephelometer (Siemens Health AG).

### In vitro study

#### Cells

Primary human umbilical vein endothelial cells (HUVECs) were isolated from freshly collected umbilical cords using a standard technique and grown in endothelial cell growth medium with endothelial cell growth supplement (C-22010, Promo Cell, Heidelberg, Germany), in a 5% CO_2_ incubator at 37 °C, until the experimental usage. The use of human material was approved by the Ethics Committee of the Faculty of Medicine at the University of Erlangen‐Nürnberg (case no. 21-331-B). All subjects enrolled in this research have given an informed consent according to the ethical guidelines. In all experiments, HUVECs at passage 1–2 were used.

Human monocytic cell line THP-1 cells from acute monocytic leukemia patient (ACC 16, DSMZ-German Collection of Microorganisms and Cell Cultures GmbH) were cultured in very low endotoxin RPMI media (Biochrom, Berlin, Germany) containing 1% penicillin-streptamycin, 1% l-glutamine (2 mM and 10% fetal calf serum (Biochrom), in 5% CO_2_ incubator at 37 °C, until the experimental usage.

#### Analysis of cell viability

Viability of HUVECs was measured using Gallios cytofluorometer™ (Beckman Coulter, Fullerton, CA, USA). Briefly, 10 × 10^4^ cells/well were seeded into 12-well plates and samples were incubated at the respective temperature (37 °C, 25 °C or 4 °C) for 3 h or 24 h. Following the incubation process, cells were harvested and 50 µl of single cell suspensions were incubated with 250 µl of staining solution for 30 min at 37 °C. For 1 ml staining solution, 10 μg Hoechst 33342, 25 ng AxV-FITC and 66.6 ng PI (propidium iodide) were added to Ringer solution. Samples were analyzed using flow cytometry with the following laser setting: FITC fluorescence was detected with the FL1 sensor (525/38 nm band pass filter, BP); Hoechst 33342 fluorescence was detected at 405 nm using the FL9 sensor (430/40 nm BP) and the PI fluorescence was detected with the FL3 sensor (620/30 nm BP). Electronic compensation was used to eliminate fluorescence bleed-through. Data analysis was carried out with the Kaluza software Version 1.2 (Beckman Coulter).

#### Analysis of THP-1 recruitment and fluorescent cell staining

HUVECs were seeded in the Ibidi^®^ µ-slides (Ibidi, Munich, Germany) at the final concentration of 70 × 10^4^ cells/ml and incubated for 2 days to achieve the complete cell coverage of the slide surface. Afterwards, HUVEC samples and THP-1 cell suspensions were placed at the respective temperature and incubated for 3 h or 24 h. After the incubation periods, THP-1 cell suspensions with the concentration of 75 × 10^4^ cells/ml were prepared and seeded onto HUVECs. Samples were incubated for another hour at the respective temperature. At the end of the incubation period, slides were washed with media three times to remove the non-adherent THP-1 cells. Subsequently, the samples were fixed with 4% buffered paraformaldehyde (Roth GmbH, Karlsruhe, Germany) for 15 min and permeabilized with 0.2% Triton X-100 (Sigma-Aldrich, Munich, Germany) for 5 min. Nuclei were stained using Hoechst 33342 at a final concentration of 5 μg/ml and F-actin filaments were stained with Alexa 488-phalloidin (Invitrogen, Thermo Fisher Scientific GmbH, Dreieich, Germany). Staining was visualized using fluorescence microscope Zeiss Axio Observer Z1 (Zeiss, Jena, Germany) at 10 × and 20 × magnification. The numbers of recruited THP-1 cells were counted using Image J.

### Statistical analysis

The data analysis was performed with the Statistica 13.1 software. The statistical significance of the difference between the measurements before and after CWI was verified using Student’s t-test for dependent groups and its non-parametric alternative, the Wilcoxon signed-rank test (when distributions deviated from normal). Spearman rank-order correlation test was used to analyze the correlations between the different parameters. All p-values are two-tailed and the statistical significance was set at the value of p ≤ 0.05.

The differences between the in vitro study groups were compared using unpaired or Kruskal–Wallis test. Every in vitro experiment was repeated three times and all samples were compared to the control group kept at 37 °C, unless specified otherwise. The differences in cell viability between groups were compared using one-way ANOVA with multiple comparisons and the Kruskal–Wallis test. The differences in THP-1 cell recruitment were assessed using an unpaired Student’s t-test. P values indicated in the graphs are as follows: *p < 0.1, **p < 0.01, ***p < 0.001, and ****p < 0.0001. The statistical analyses and in vitro data presentation were done using GraphPad Prism.

### Institutional review board statement

The study was conducted in accordance with the tenets of the Declaration of Helsinki and approved by the Ethics Committee of the Regional Medical Chamber in Krakow, Poland (approval No.: 212/KBL/OIL/2022). The use of human cells was approved by the Ethics Committee of the Faculty of Medicine at the University of Erlangen‐Nürnberg (case no. 21-331-B).

### Informed consent statement

Informed consent was obtained from all subjects involved in the study.

## Results

### Effects of single CWI in healthy subjects

The study group consisted of 13 male volunteers with the mean age of 21–25 years, body mass of 69–88 kg, and body height of 170–187 cm. No statistically significant changes in blood pressure were observed between before and after CWI, but the heart rate was significantly reduced by CWI (Table 1), and the subjects’ body temperature decreased significantly from 36.45 °C before immersion to 31.55 °C (moderate hypothermia) after immersion (p = 0.003).

**Table 1:** Mean values (± standard deviation) of blood pressure, heart rate, and body temperature before and after CWI.

Parameter	Baseline (n = 13)	After cold water immersion (n = 13)	p (dependent)
Systolic blood pressure (mmHg)	130.31 ± 5.07	131.85 ± 11.07	0.5756
Diastolic blood pressure (mmHg)	73.92 ± 7.35	76.54 ± 11.77	0.4132
Heart rate (beats/min)	68.23 ± 11.24	61.62 ± 12.49	0.0032**
Body temperature (°C)	36.45 ± 0.40	31.55 ± 2.87	0.003**

**p < 0.01.

**Figure 1:**
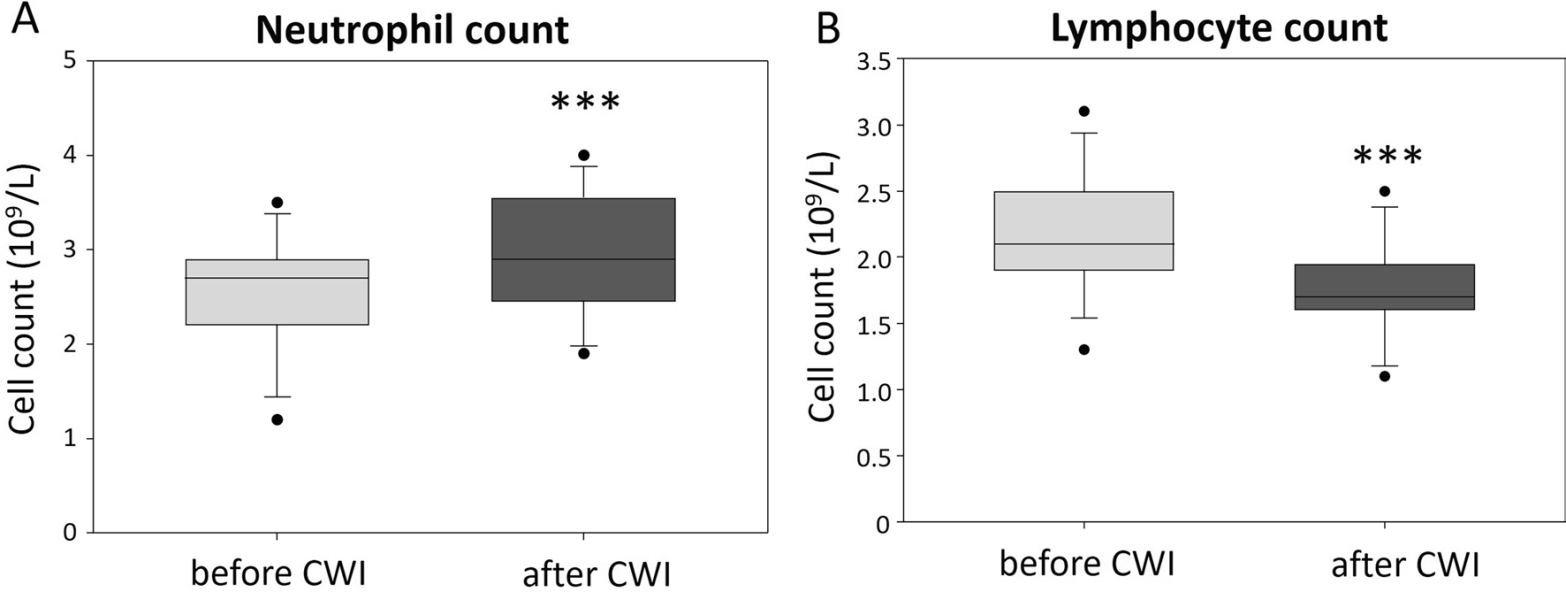
The effect of CWI on (**A**) neutrophil count and (**B**) lymphocyte count in young healthy males. Graphs show the median, 10th, 25th, 75th, and 90th percentile. The statistical differences between the regions were calculated using Student’s t-test, n = 13; ***p < 0.001.

The majority of morphological parameters remained unchanged after CWI. However, a statistically significant increase in MCV was observed, as well as a significant increase in the neutrophil and a reduction in lymphocyte counts after CWI (Fig. 1A,B). The detailed complete blood count results are presented in Table 2. No significant correlations between body temperature after CWI and the morphological parameters were detected. The significant increase in MCV following CWI was not correlated with reticulocyte count in blood (Supplementary Fig. 1A).

**Table 2:** Mean values (± standard deviation) of the morphological parameters before and after CWI.

Parameter	Baseline (n = 13)	After cold water immersion (n = 13)	p (dependent)
WBC (10^9^/l)	5.44 ± 14.91	5.36 ± 0.79	0.5973
RBC (10^12^/l)	5.02 ± 0.22	5.05 ± 0.18	0.7013
HGB (g/dl)	14.95 ± 0.72	14.97 ± 0.55	0.8597
HCT (%)	45.95 ± 1.65	45.92 ± 1.19	0.2235
MCV (fl)	90.70 ± 3.19	91.36 ± 3.03	0.00076***
MCH (pg)	29.81 ± 1.30	29.79 ± 1.28	0.8125
MCHC (g/dl)	32.86 ± 0.62	32.59 ± 0.52	0.0899
RET (× 10^9^/l)	81.85 ± 14.91	86.57 ± 19.36	0.2133
RDW-SD (fl)	12.52 ± 0.44	12.54 ± 0.44	0.3581
PLT (10^9^/l)	243.62 ± 64.63	249.23 ± 64.44	0.3068
PDW (fl)	55.55 ± 7.45	56.88 ± 6.12	0.3708
MPV (fl)	8.45 ± 1.01	8.52 ± 0.94	0.4893
PCT (%)	0.20 ± 0.04	0.21 ± 0.04	0.0935
NEU (10^9^/l)	2.55 ± 0.61	2.92 ± 0.64	0.00017***
LYM (10^9^/l)	2.20 ± 0.44	1.75 ± 0.36	0.00013***
MONO (10^9^/l)	0.42 ± 0.10	0.42 ± 0.06	0.5293
EOS (10^9^/l)	0.16 ± 0.08	0.15 ± 0.08	0.1797
BASO (10^9^/l)	0.03 ± 0.05	0.04 ± 0.05	0.5929

***p < 0.001.

**Figure 2:**
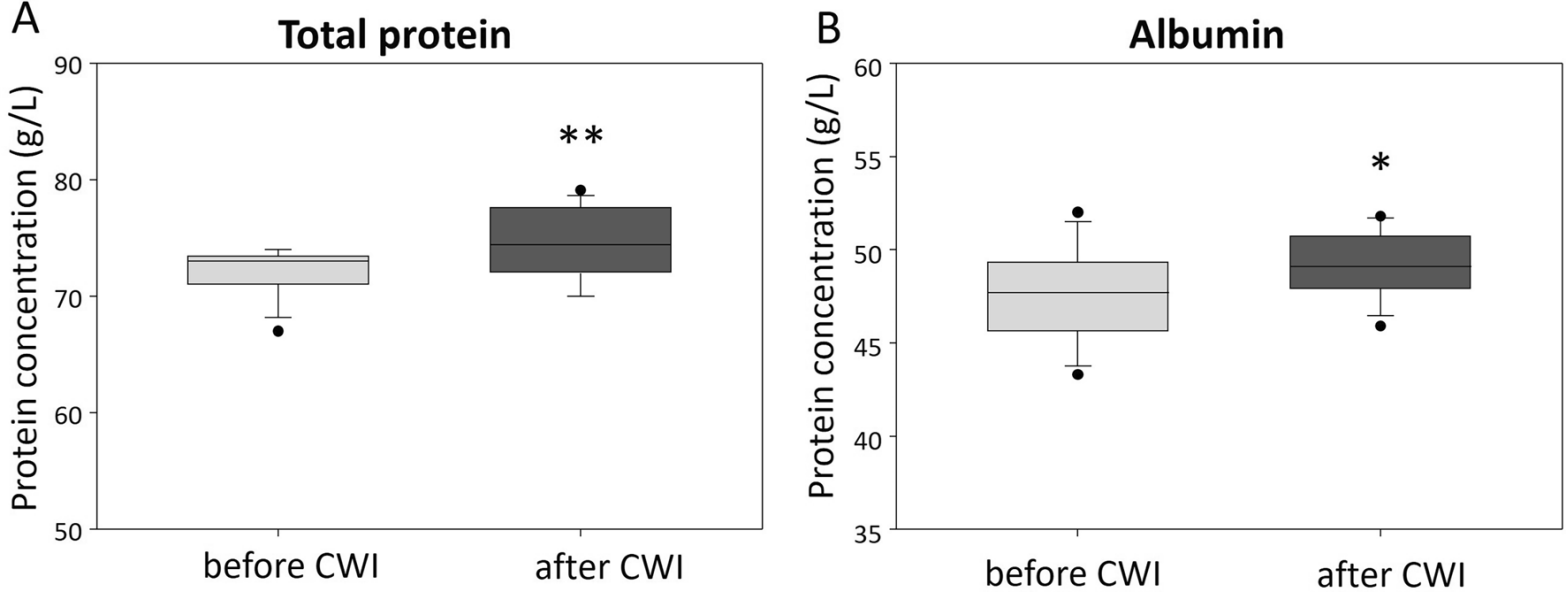
The effect of CWI on (**A**) total protein and (**B**) albumin concentrations in the blood of young healthy males. Graphs show the median, 10th, 25th, 75th, and 90th percentile. The statistical differences between the regions were calculated using Student’s t-test, n = 13; *p < 0.05, **p < 0.01.

The evaluation of biochemical parameters indicated that statistically significant increase in total protein and albumin concentration occurred post-CWI, as illustrated in Table 3 and Fig. 2. As expected, there was a positive correlation between total protein and albumin concentration (Supplementary Fig. 1B). Other parameters, including the concentrations of hs-CRP, fibrinogen and globulins were not significantly changed by CWI, although a slight tendency towards increased alpha-2 globulin was present (p = 0.076).

**Table 3:** Mean values (± standard deviation) of selected biochemical parameters before and after CWI.

Parameter	Baseline (n = 13)	After cold water immersion (n = 13)	p (dependent)
hs-CRP (mg/l)	0.66 ± 0.89	0.68 ± 0.95	0.6121
FIBR (g/l)	2.30 ± 0.49	2.27 ± 0.40	0.7821
Total protein (g/l)	72.08 ± 2.06	74.84 ± 2.96	0.0056**
Albumin (g/l)	47.56 ± 2.59	49.28 ± 1.77	0.0224*
Alpha-1 globulin (g/l)	2.54 ± 0.38	2.62 ± 0.41	0.2014
Alpha-2 globulin (g/l)	5.32 ± 0.87	5.79 ± 0.68	0.0756
Beta-1 globulin (g/l)	3.78 ± 0.39	3.97 ± 0.43	0.1774
Beta-2 globulin (g/l)	3.23 ± 0.92	3.18 ± 0.43	0.8052
Gamma globulins (g/l)	9.58 ± 1.21	9.94 ± 1.49	0.1886
A/G ratio	1.97 ± 0.28	1.95 ± 0.19	0.8013

*p < 0.05, **p < 0.01.

EI, the rheological indicator of erythrocyte deformability, exhibited a statistically significant reduction after CWI at the following shear stress values: 2.19, 4.24, 8.23, 15.59, 30.94, and 60.00 Pa (Table 4). As shown in Supplementary Fig. 2, there was a significant negative correlation between MCV after CWI and the EI values at 15.59 Pa and 30.94 Pa.

**Table 4:** Mean values (± standard deviation) of the elongation index before and after CWI.

Parameter	Baseline (n = 13)	After cold water immersion (n = 13)	p (dependent)
EI at 0.30 Pa	0.049 ± 0.009	0.047 ± 0.007	0.4017
EI at 0.58 Pa	0.151 ± 0.014	0.143 ± 0.011	0.1173
EI at 1.13 Pa	0.237 ± 0.015	0.231 ± 0.012	0.1296
EI at 2.19 Pa	0.341 ± 0.01	0.332 ± 0.009	0.0099**
EI at 4.24 Pa	0.433 ± 0.01	0.428 ± 0.001	0.0444
EI at 8.23 Pa	0.513 ± 0.007	0.505 ± 0.008	0.0041
EI at 15.59 Pa	0.566 ± 0.007	0.559 ± 0.008	0.0006***
EI at 30.94 Pa	0.605 ± 0.006	0.598 ± 0.008	0.0002***
EI at 60.00 Pa	0.630 ± 0.005	0.624 ± 0.006	0.0025**

* p < 0.1, ** p < 0.01, *** p < 0.001.

In contrast to reduced erythrocyte deformability, the assessment of aggregation parameters before and after CWI demonstrated that CWI had no significant effect on aggregation-related factors (Table 5).

**Table 5:** Mean values (± standard deviation) of erythrocyte aggregation parameters before and after CWI.

Parameter	Baseline (n = 13)	After cold water immersion (n = 13)	p (dependent)
AMP (au)	38.10 ± 2.34	36.95 ± 3.21	0.2794
T_1/2_ (s)	4.14 ± 0.95	4.27 ± 1.24	0.7663
AI (%)	49.85 ± 4.97	49.59 ± 6.73	0.9099

### Effects of lowered temperature on human endothelial cells

**Figure 3:**
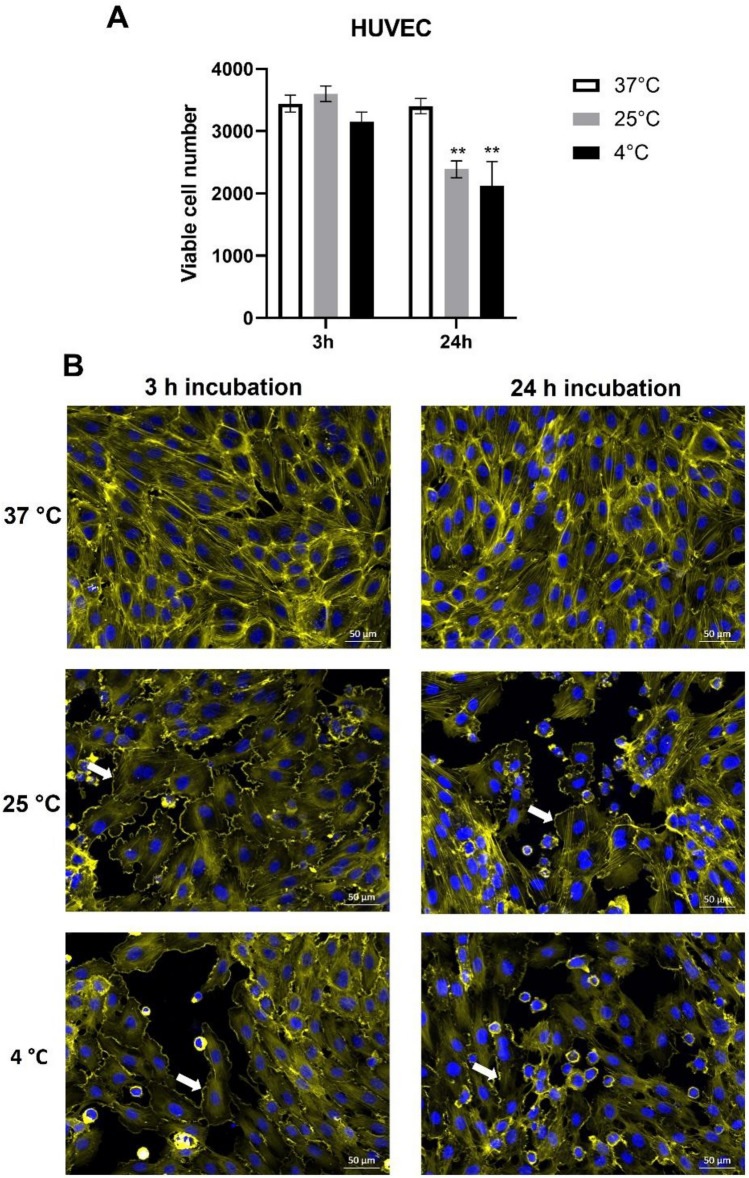
Effect of low temperature on endothelial cell viability and morphology. (**A**) HUVECs were incubated at the respective temperatures for 3 h or 24 h and the numbers of viable cells was determined by flow cytometry. Data of 3 independent experiments are shown; **p < 0.01. (**B**) Cell morphology was visualized using F-actin and nuclear staining, and fluorescent microscopy. White arrows indicate membrane ruffles at lowered temperature.

To investigate whether the temperature decrease to 25 °C, corresponding to 3rd grade (severe) hypothermia in humans, affects the behavior of human endothelial cells, an in vitro study was performed. The results showed that incubation of cells at 25 °C for 3 h did not affect the cell number compared to HUVECs grown at 37 °C, indicating that 3 h temperature reduction had no effect on cell viability (Fig. 3A). However, a striking effect of lowered temperature on cell morphology was noted, as the membrane ruffles appeared in HUVECs exposed to 25 °C and cell–cell contacts were reduced, in parallel with re-arrangement of F-actin cytoskeleton. As shown in Fig. 3B, F-actin was distributed in form of cell-spanning fibers, but the majority of fibers were localized along the cell membranes, aligning with cell junctions at 37 °C. At 25 °C, the actin fibers lining the cell membranes were lost and cells started to shrink slightly.

**Figure 4:**
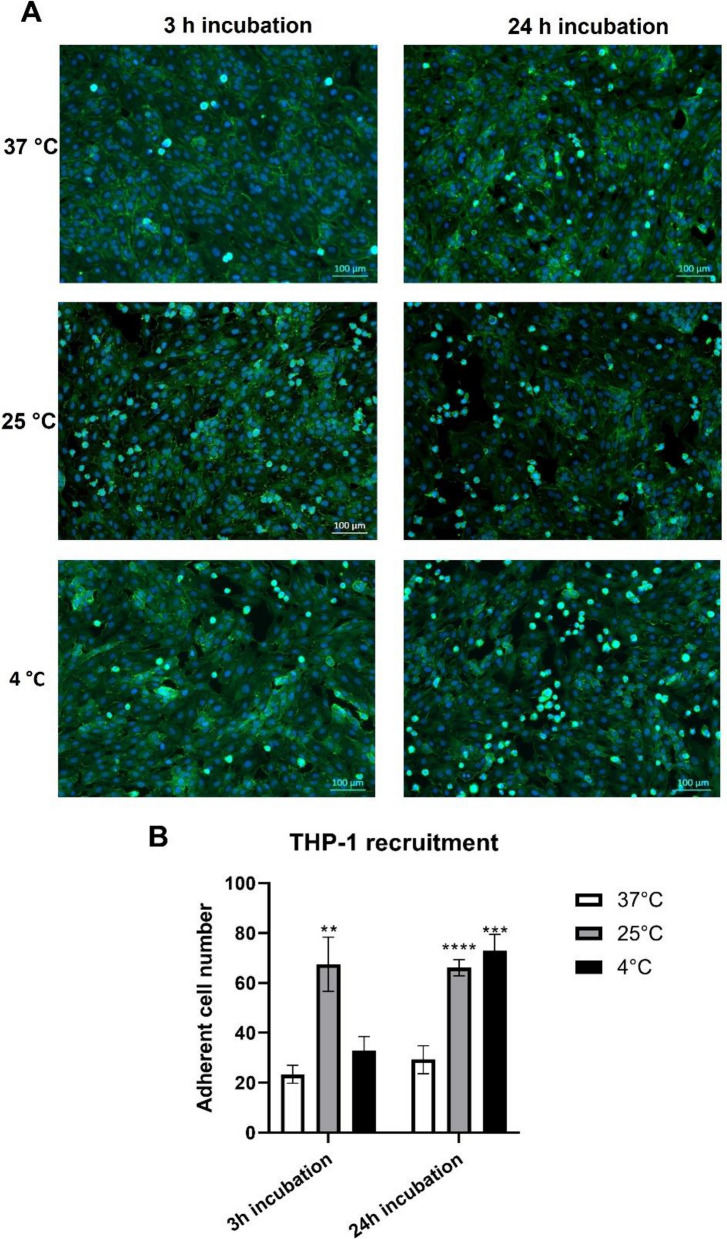
Effect of low temperature on THP-1 recruitment by endothelial cells. HUVECs and THP-1 monocytic cells were incubated at the respective temperatures for 3 h or 24 h. (**A**) Images of attached THP-1 cells were taken using fluorescent microscopy. (**B**) Data quantification shows the numbers of adherent cells per image (61.46 mm^2^) from 3 independent experiments. **p < 0.01, ***p < 0.001, and ****p < 0.0001.

Moreover, a significant increase in numbers of THP-1 monocytic cell recruited by HUVECs grown at 25 °C for 3 h was observed (Fig. 4), indicating endothelial cell activation at lowered temperature. Interestingly, although a further reduction of temperature to 4 °C led to similar changes in cell morphology as observed at 25 °C, it did not induce comparable adhesion of THP-1 cells. After 24 h of cold temperature treatment, no significant differences between HUVECs incubated at 25 °C and 4 °C were observed (Figs. 3 and 4).

## Discussion

The analysis of the blood haemorheological parameters together with blood morphological and biochemical indicators in the male subjects exposed to a single non-life-threatening CWI provides valuable information about the nature of the physiological responses to short-term CWI. While the majority of the parameters were not affected by the single immersion, several significant changes were detected in both rheological and biochemical parameters.

In the regular winter swimmers, erythrocyte deformability plays an important role in facilitating blood flow not only during the winter swimming season, but also after the season^19,20^. However, the present study revealed that upon a significant decrease in body temperature after a single CWI, the EI, an erythrocyte deformability measure which refers to erythrocyte length changes in relation to width while stretched, was reduced at the shear stress values between 2.19 Pa and 60.00 Pa. The unfavourable changes in erythrocyte deformability may result in a further reduction in blood flow through the vasculature already constricted by cooling. In a previous study by Teległów et al.^21^, an increase in the EI at shear stress values of 0.30–4.24 Pa was observed in a single session of maximum effort performed in water with a temperature of 4 °C, in the absence of simultaneous changes in erythrocyte aggregation, plasma viscosity, or erythrocyte membrane fatty acid composition. This indicates that while the static immersion has a negative effect on erythrocyte deformability, active effort improves circulation and prevents unfavourable changes in cell deformability.

The erythrocyte deformability is influenced by their membrane properties, but also by blood viscosity, which depends on the plasma viscosity and the ratio of erythrocyte volume to blood plasma, i.e. the haematocrit value^22^. In the present study, apart from an increase in MCV no changes in blood haematocrit or other erythrocyte system indicators were observed. However, the elevated MCV could be related to the severity of blood flow disruption under the impact of a single CWI, as we observed a negative correlation between MCV values and erythrocyte deformability at higher shear stress range. It must also be noted that overestimated values of MCV are commonly obtained in the presence of an increased number of young erythrocytes. However, no statistically significant changes in reticulocyte numbers were detected in the present study upon CWI, and MCV values did not correlate with reticulocyte count, which confirms that the alterations in MCV observed among the male subjects are solely due to a single immersion.

The rheological properties of blood mainly result from the properties of erythrocyte, which strongly affect the blood flow in vessels, but also from the properties of plasma, including the plasma proteins. In particular, fibrinogen concentration has a strong effect on the hemorheological parameters, as its increase induces erythrocyte aggregation, leading to a higher blood viscosity^23^. In the present study, however, no changes in fibrinogen concentrations or in erythrocyte aggregation parameters (AI, AMP, T_1/2_) were observed. Other factors that influence the formation of erythrocyte aggregates include lipoproteins, macroglobulins, immunoglobulins, haematocrit, erythocyte shape and deformability, as well as cell membrane surface properties^23^. Our study did not reveal statistically significant changes in alpha-1 globulin, alpha-2 globulin, beta-1 globulin, gamma globulins, or A/G ratio. In contrast, increased concentrations of total protein and albumin were observed after CWI, which most likely resulted from the short-term response of the subjects to CWI, without prior acclimatization to the extreme conditions.

According to Jansky et al.^16^, a single CWI at 14 °C for 1 h had a marginal impact on the immune system of athletic young males, monitored immediately after the immersion. As CWIs continued (3 times a week for 6 weeks), a small but significant increase in the proportion of monocytes and lymphocytes was reported. In the present study, lymphocyte count was decreased, while the neutrophil count was increased, but both values stayed within laboratory norms. Presumably, both the extent (within the physiological range) and the differing direction of changes in the post-stress indicators studied here reflect a response of the participants to the single CWI exposure.

Hypothermia conditions may also affect the cells of vascular system^24,25^. Our in vitro results indicate that the exposure of primary HUVECs to the lowered temperature (25 °C) for 3 h affects their morphology and function, leading to an increased recruitment of THP-1 monocytic cells. A similar effect was observed in cells grown at 4 °C, but only after 24 h, which indicates that the moderate lowering of temperature leads to faster cell activation. Collectively, these data contribute to our understanding of the physiological processes induced by extreme cooling in cells of the vascular system.

## Conclusions

A single CWI does not affect the majority of blood parameters in young healthy males, but may worsen erythrocyte deformability and increase the total protein concentration. The observed effect most likely represents a short-term response of the subjects to CWI-related physiological stress, which results from the lack of acclimatization to the extreme cold treatment.

### Supplementary Information

Supplementary Figures.

## Data Availability

The datasets used and/or analysed during the current study are available from the corresponding author on reasonable request.

## Abbreviations

AI: Erythrocyte aggregation index
AMP: Erythrocyte aggregation amplitude
BASO: Basophil concentration
CRP: C-reactive protein
CWI: Cold water immersion
EI: Erythrocyte elongation index
EOS: Eosinophil concentration
FIBR: Fibrinogen
HCT: Haematocrit
HGB: Haemoglobin
LYM: Lymphocyte concentration
MCH: Mean corpuscular haemoglobin
MCHC: Mean corpuscular haemoglobin concentration
MCV: Mean corpuscular volume
MON: Monocyte concentration
NEU: Neutrophil concentration
PCT: Plateletcrit
PDW: Platelet distribution width
PLT: Platelet concentration
RBC: Red blood cells (erythrocytes)
RDW-SD: Red blood cell distribution width standard deviation
T_1/2_: Half-time of total erythrocyte aggregation
WBC: White blood cells
