# Supplementary material for: Effect of a single immersion in cold water below 4 °C on haemorheological properties of blood in healthy men
Source: Sci Rep. 2024 Apr 12;14:8554. doi: 10.1038/s41598-024-58731-2 (PMC11015000; doi:10.1038/s41598-024-58731-2)
Supplement: Supplementary file 1 — Supplementary Figures. [file 41598_2024_58731_MOESM1_ESM.docx]

**SUPPLEMENTARY MATERIAL**

**Effect of a single immersion in cold water below 4°C on haemorheological properties of blood in healthy men**

Aneta Teległów^1^*, Hatice Genç^2^, Iwona Cicha^2^

^1^ Department of Health Promotion, Institute of Basic Sciences, University of Physical Education in Krakow, 31-571 Krakow, Poland

^2^ Cardiovascular Nanomedicine Unit, Section of Experimental Oncology and Nanomedicine (SEON), Department of Otorhinolaryngology, Head and Neck Surgery, Universitätsklinikum Erlangen, Germany

*Corresponding author: aneta.teleglow@awf.krakow.pl


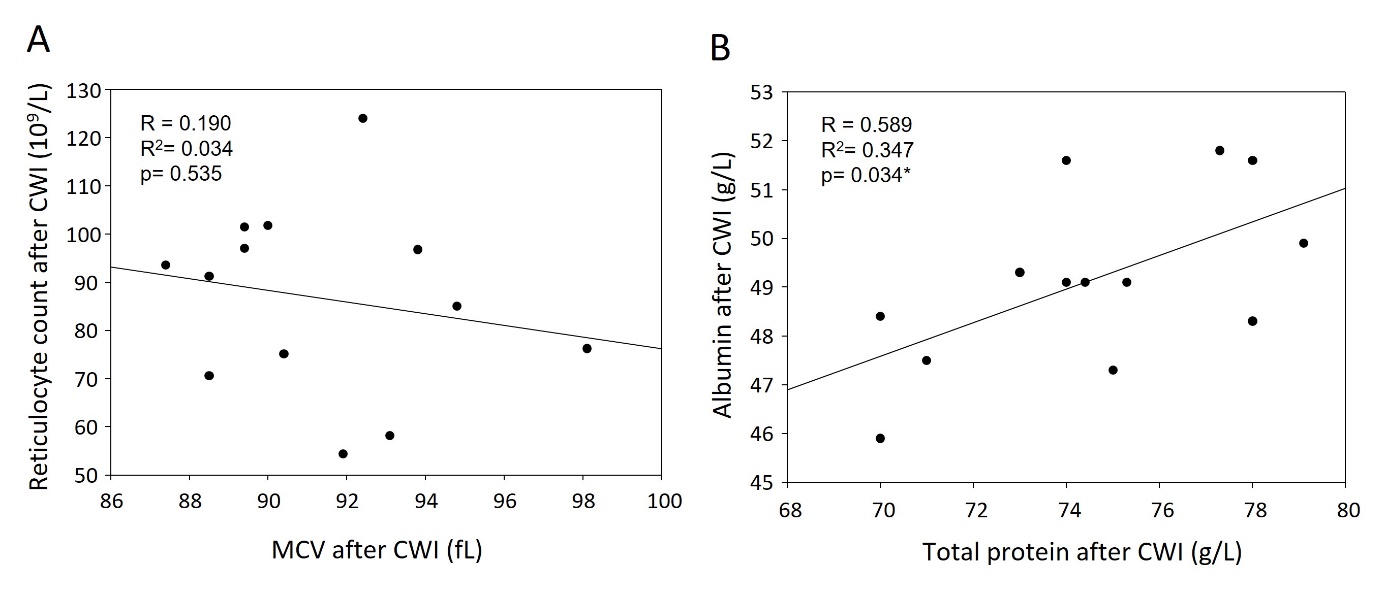


***Suppl. Figure 1****. Correlations of morphological (A) and biochemical blood parameters (B) after CWI. (A) No significant correlation between MCV and reticulocyte count; (B) Significant positive correlation between albumin concentration and total blood protein. Spearman rank-order correlation test was used to analyze the correlations.*


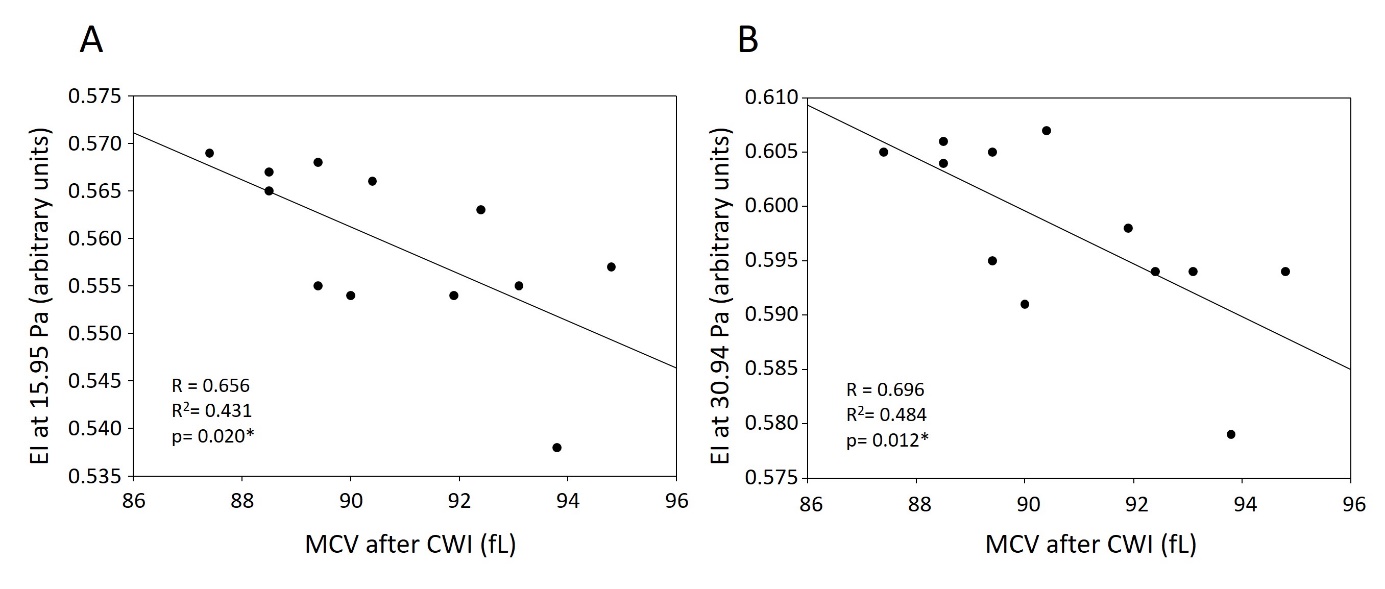


***Suppl. Figure 2****. Significant negative correlations of MCV with EI at 15.95 Pa (A) and 30.94 Pa (B). Spearman rank-order correlation test was used to analyze the correlations.*
